# Quantum interference effect in electron tunneling through a quantum-dot-ring spin valve

**DOI:** 10.1186/1556-276X-6-265

**Published:** 2011-03-28

**Authors:** Jing-Min Ma, Jia Zhao, Kai-Cheng Zhang, Ya-Jing Peng, Feng Chi

**Affiliations:** 1 Department of Physics, Bohai University, Jinzhou 121000, China

## Abstract

Spin-dependent transport through a quantum-dot (QD) ring coupled to ferromagnetic leads with noncollinear magnetizations is studied theoretically. Tunneling current, current spin polarization and tunnel magnetoresistance (TMR) as functions of the bias voltage and the direct coupling strength between the two leads are analyzed by the nonequilibrium Green's function technique. It is shown that the magnitudes of these quantities are sensitive to the relative angle between the leads' magnetic moments and the quantum interference effect originated from the inter-lead coupling. We pay particular attention on the Coulomb blockade regime and find the relative current magnitudes of different magnetization angles can be reversed by tuning the inter-lead coupling strength, resulting in sign change of the TMR. For large enough inter-lead coupling strength, the current spin polarizations for parallel and antiparallel magnetic configurations will approach to unit and zero, respectively.

PACS numbers:

## Introduction

Manipulation of electron spin degree of freedom is one of the most frequently studied subjects in modern solid state physics, for both its fundamental physics and its attractive potential applications [1,2]. Spintronics devices based on the giant magnetoresistance effect in magnetic multi-layers such as magnetic field sensor and magnetic hard disk read heads have been used as commercial products, and have greatly influenced current electronic industry. Due to the rapid development of nanotechnology, recent much attention has been paid on the spin injection and tunnel magnetoresistance (TMR) effect in tunnel junctions made of semiconductor spacers sandwiched between ferromagnetic leads[3]. Moreover, semiconductor spacers of InAs quantum dot (QD), which has controllable size and energy spectrum, has been inserted in between nickel or cobalt leads[4-6]. In such a device, the spin polarization of the current injected from the ferromagnetic leads and the TMR can be effectively tuned by a gate nearby the QD, and opens new possible applications. Its new characteristics, for example, anomalies of the TMR caused by the intradot Coulomb repulsion energy in the QD, were analyzed in subsequent theoretical work based on the nonequilibrium Green's function method [7].

The TMR is a crucial physical quantity measuring the change in system's transport properties when the angle *φ *between magnetic moments of the leads rotate from 0 (parallel alignment) to arbitrary value (or to *φ *in collinear magnetic moments case). Much recent work has been devoted to such an effect in QD coupled to ferromagnetic leads with either collinear[4-13] or noncollinear[14-16] configurations. It was found that the electrically tunable QD energy spectrum and the Coulomb blockade effect dominate both the magnitude and the signs of the TMR[4-16].

On the other hand, there has been increasing concern about spin manipulation via quantum interference effect in a ring-type or multi-path mesoscopic system, mainly relying on the spin-dependent phase originated from the spin-orbit interaction existed in electron transport channels[17-20]. Many recent experimental and theoretical studies indicated that the current spin polarization based on the spin-orbital interaction can reach as high as 100%[21-23] or infinite[24-29]. Meanwhile, large spin accumulation on the dots was realized by adjusting external electrical field or gate voltages to tune the spin-orbit interaction strength (or equivalently the spin-dependent phase factor)[27-30]. Furthermore, there has already been much very recent work about spin-dependent transport in a QD-ring connected to collinear magnetic leads[31-34]. Much richer physical phenomena, such as interference-induced TMR enhancement, suppression or sign change, were found and analyzed[31-34].

**Figure 1:**
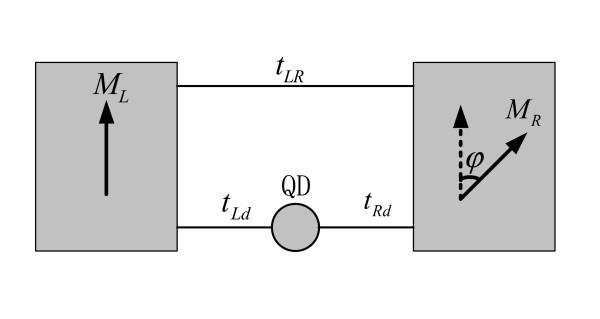
**Schematic picture of single-dot ring with noncollinearly polarized ferromagnetic leads**.

Up to now, the magnetic configurations of the leads coupled to the QD-ring are limited to collinear (parallel and antiparallel) one. To the best of our knowledge, transport characteristics of a QD-ring with noncollinear magnetic moments have never studied, which is the motivation of the present paper. As shown in Figure 1 we study the device of a quantum ring with a QD inserted in one of its arms. The QD is coupled to the left and the right ferromagnetic leads whose magnetic moments lie in a common plane and form an arbitrary angle with respect to each other. There is also a bridge between the two leads indicating inter-lead coupling. It should be noted that such a QD-ring connected to normal leads has already been realized in experiments[35-40]. Considering recent technological development[4-6], our model may also be realizable.

## Model and Method

The system can be modeled by the following Hamiltonian[14,20,30](1)

where  is the creation (annihilation) operator of the electrons with momentum *k*, spin-*σ *and energy *ε_kβσ _*in the *β*th lead (*β *= *L, R*);  creates (annihilates) an electron in the QD with spin *σ *and energy *ε_d_*; *t_βd _*and *t_LR _*describe the dot-lead and inter-lead tunneling coupling, respectively; *U *is the intradot Coulomb repulsion energy. *φ *denotes the angle between the magnetic moments of the leads, which changes from 0 (parallel alignment) to *π *(antiparallel alignment).

The current of each spin component flowing through lead *β *is calculated from the time evolution of the occupation number , and can be written in terms of the Green's functions as[20,30](2)

where the Keldysh Green's function *G*(*ε*) is the Fourier transform of *G*(*t *- *t'*) defined as , . In our present case, it is convenient to write the Green's function as a 6 *× *6 matrix in the representation of (|*L *↑〉, |*R *↓〉, |*d *↑〉, |*L *↓〉, |*R *↓〉, |*d *↓〉). Thus the lesser Green's function *G^ <^*(*ε*) and the associated retarded (advanced) Green's function *G*^*r*(*a*)^(*ε*) can be calculated from the Keldysh and the Dayson equations, respectively. Detail calculation process is similar to that in some previous works [20,30], and we do not give them here for the sake of compactness. Finally, the ferromagnetism of the leads is considered by the spin dependence of the leads' density of states *ρ*_*βσ*_. Explicitly, we introduce a spin-polarization parameter for lead *β *of *P_β _*= (*ρ*_*β*↑ _- *ρ*_*β↓*_)/(*ρ*_*β*↑ _+ *ρ*_*β*↓_), or equivalently, *ρ*_*β*↑(↓) _= *ρ*_*β *_(1 ± *P*_*β*_), with *ρ*_*β *_being the spin-independent density of states of lead *β *.

## Result and Discussion

In the following numerical calculations, we choose the intradot Coulomb interaction *U *= 1 as the energy unit and fix *ρ_L _*= *ρ_R _= ρ*_0 _= 1, *t_Ld _*= *t_Rd _*= 0.04. Then the line-width function in the case of *p_L _= p_R _= *0 is Γ*_β _*≡ 2π*ρ_β_*|*t_βd_*|^2 ^≈ 0.01, which is accessible in a typical QD[41-43]. The bias voltage *V *is related to the left and the right leads' chemical potentials as *eV *= *μ_L _*- *μ_R_*, and *μ_R _*is set to be zero throughout the paper.

**Figure 2:**
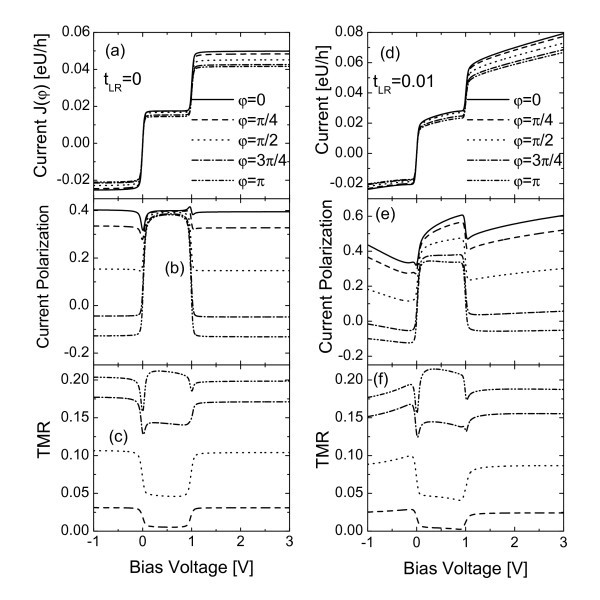
**Total current *J*, current spin polarization *p *and TMR each as a function of the bias voltage for different values of *φ*. *t_LR _*= 0 in Figs. (a) to (c) and *t_LR _*= 0.01 in Figs. (d) to (f)**. The other parameters are intradot energy level *ε_d _*= 0, temperature *T *= 0.01, and polarization of the leads *P_L _*= *P_R _*= 0.4.

Bias dependence of electric current *J *= *J*_↑ _+ *J*↓, where *J_σ _*= (*J_Lσ _*- *J_Rσ_*)/2 is the symmetrized current for spin-*σ*, current spin polarization *p *= (*J*_↑ _*- J*↓)*/*(*J*_↑ _+ *J*↓), and TMR=[*J*(*φ = *0) - *J*(*φ)*]/*J*(*φ) *are shown in Figure 2 for selected values of the angle *φ*. In the absence of inter-lead coupling (*t_LR _*= 0), the electric current in Figure 2(a) shows typical step configuration due to the Coulomb blockade effect. The current step emerged in the negative bias region occurs when the dot level *ε_d _*is aligned to the Fermi level of the right lead (*μ_R _*= 0). Now electrons tunnel from the right lead via the dot to the left lead because *μ_L _*= *eV < εε_d _*= 0. The dot can be occupied by a single electron with either spin-up or spin-down orientation, which prevents double occupation on *ε_d _*due to the Pauli exclusion principle. Since the other transport channel *ε_d _*+ *U *is out of the bias window, the current keeps as a constant in the bias regime of *eV *<*ε_d _*= 0. In the positive bias regime of *ε_d _*<*eV *<*ε_d _*+ *U *a single electron transport sequentially from the left lead through the dot to the right lead, inducing another current step. The step at higher bias voltage corresponds to the case when *ε_d _*+ *U *crosses the Fermi level. Now the dot may be doubly occupied, and no step will emerge regardless of the increasing of the bias voltage.

When the relative angle between the leads' magnetic moments *φ*. rotates from 0 to *π*, a monotonous suppression of the electric current appears, which is known as the typical spin valve effect. The suppression of the current can be attributed to the increased spin accumulation on the QD[14-16]. Since the line-width functions of different spin orientations are continuously tuned by the angle variation, a certain spin component electron with smaller tunneling rate will be accumulated on the dot, and furthermore prevents other tunnel processes. As shown in Figure 2(b), the current spin polarizations in the bias ranges of *eV *<*ε_d _*and *eV *>*ε_d _*+ *U *are constant and monotonously suppressed by the increase of the angle, which changes the spin-up and spin-down line-width functions. In the Coulomb blockade region of *ε_d _*<*eV *<*ε_d _*+ *U*, the difference between the current spin polarizations of different values of *φ *is greatly decreased, which is resulted from the Pauli exclusion principle. The current spin polarizations also have small dips and peaks respectively near *eV *= *ε_d _*and *eV *= *ε_d _*+ *U*, where new transport channel opens. The most prominent characteristic of the TMR in Figure 2(c) is that its magnitude in the Coulomb blockade region depends much sensitively on the angle than those in other bias ranges. The deepness of the TMR valleys are shallowed with the increasing of the angle. Meanwhile, dips emerge when the Fermi level crosses *ε_d _*and *ε_d _*+ *U*. In the antiparallel configuration (*φ *= *π*), the magnitude of the TMR is larger than those in other bias voltage ranges.

When the inter-lead coupling is turned on as shown in Figure 2(d)-(f), both the studied quantities are influenced. Since the bridge between the leads serves as an electron transport channel with continuous energy spectrum, the system electric current increases with in-creasing bias voltage [Figure 2(d)]. For the present weak inter-lead coupling case of *t_LR _*<*t_βd_*, the transportation through the QD is the dominant channel with distinguishable Coulomb blockade effect. The current spin polarizations for different angles in the voltage ranges out of the Coulomb blockade one now change with the bias voltage value, but their relative magnitudes somewhat keep constant. The difference between the current spin polarization magnitude of different angle is enlarged by the interference effect brought about by the inter-lead coupling. Comparing Figure 2(f) with 2(c), the behavior of TMR is less influenced by the bridge between the leads in the present case.

**Figure 3:**
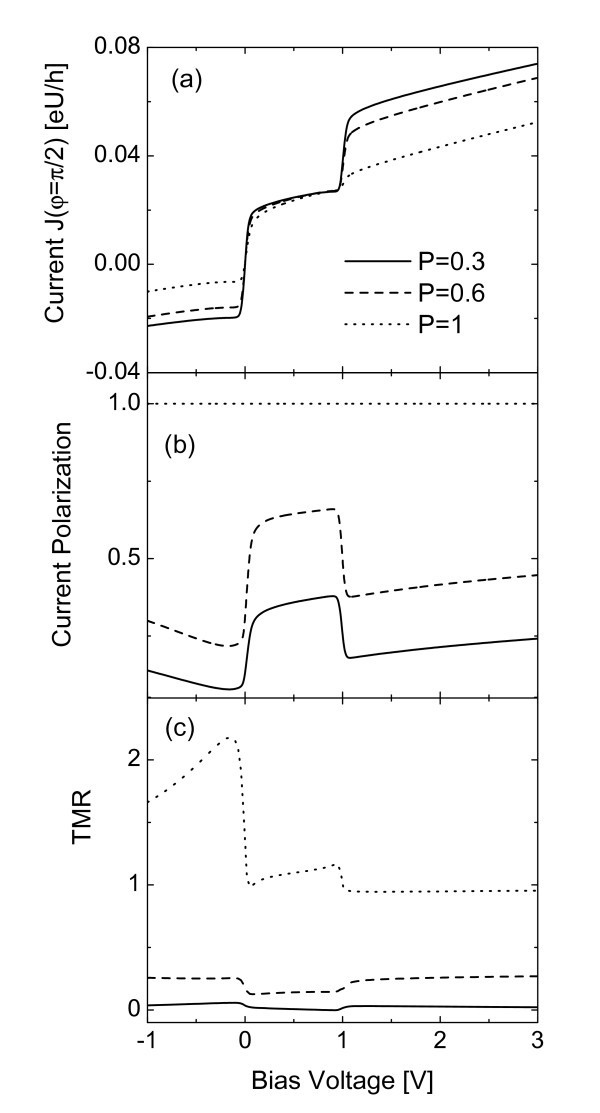
**Tunneling current, current polarization and TMR each as a function of the bias voltage for different values of leads' polarization and fixed *φ *= *π/*2**. The other parameters are as in Fig. 2.

We now fix *t_LR _*= 0.01 and the angle *φ *= *π/*2, i.e., the magnetic moments of the leads are perpendicular to each other, to examine the bias dependence of these quantities for different values of leads' polarization *P_L _*= *P_R _*= *P *. The electric currents in the bias voltage ranges of *eV *<*ε_d _*and *eV *>*ε_d _*+ *U*. are monotonously suppressed with the increase of *P *[Figure 3(a)]. This is because the spin accumulation on the dot in these bias ranges is enlarged by the increase of the leads' spin polarization. In the Coulomb blockade region, however, current magnitudes of different *P *are identical. The reason is that in this region the spin accumulation induced by the Pauli exclusion principle, which was previously discussed, plays a decisive role compared with that brought about by the leads' spin polarization. As is expected, the current spin polarization is increased with increasing *P *, which is shown in Figure 3(b). The magnitude of the TMR in Figure 3(c) increases with increasing *P*. For the half-metallic leads (*P_L _*= *P_R _*= *P *= 1), the magnitude of the TMR is much larger than those of usual ferromagnetic leads (*P_β _*< 1). All these results are similar to those of a single dot case[14-16].

**Figure 4:**
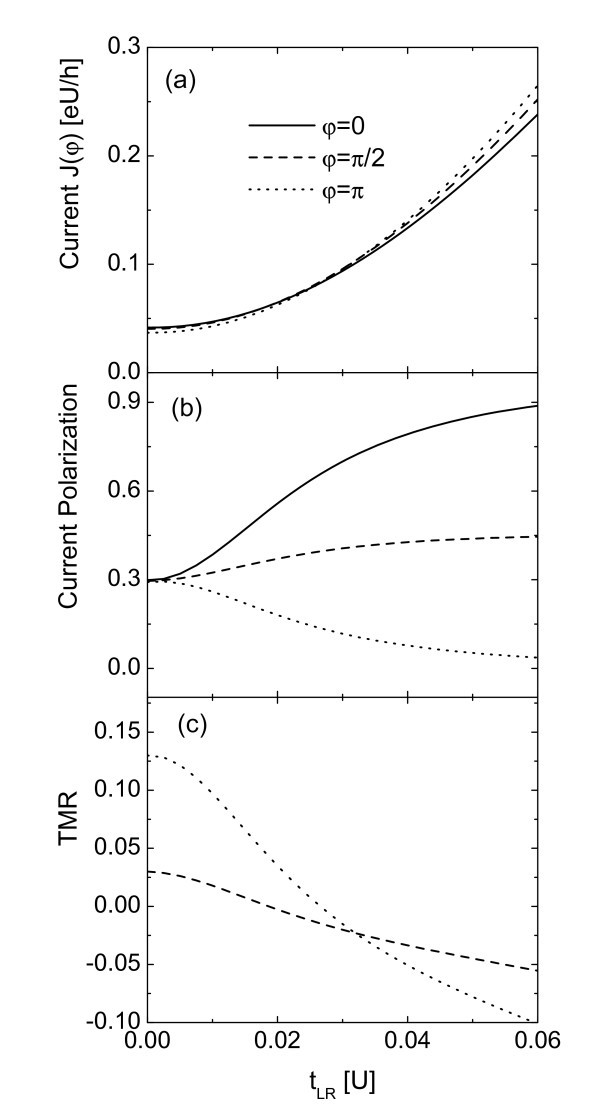
**Current, current polarization and TMR each as a function of the inter-lead coupling strength for different values of *φ *and fixed *P*_*L *_= *P*_*R *_= 0.3**. The other parameters are as in Fig. 2.

Finally we study how the inter-lead coupling strength *t_LR _*influence these quantities. In Figure 4 we show their characteristics each as a function of *t_LR _*with fixed bias voltage *eV *= *U *and *ε_d _*= 0.5, which means that we are focusing on the Coulomb blockade region. It is shown in Figure 4(a) that in the case of weak inter-lead coupling, typical spin valve effect holds true, i.e., the current magnitude is decreased with increasing *φ *as was shown in Figure 2(a) and 2(d) (see the Coulomb blockade region in them). With the increase of *t_LR_*, reverse spin valve effect is found, in other words, current magnitudes of larger angles become larger than those of smaller angles. This phenomenon can be understood by examining the spin-dependent line-width function. The basic reason is that in this Coulomb blockade region, the relative magnitudes of the currents through the QD of different angle will keep unchanged regardless of the values of *t_LR _*(see Figure 2). But the current through the bridge between the leads, which is directly proportional to the inter-lead line-width function , will be drastically varied by the angle. In the parallel configuration, for example, spin-up inter-lead line-width function  is larger than the spin-down one  since *ρ*_*L*↑ _= *ρ*_*R*↑ _= *ρ*_0 _(1 + *P*_*β*_) and *ρ*_*L*↓ _= *ρ*_*R*↓ _= *ρ*_0 _(1 - *P*_*β*_). So the current polarization will increase with increasing *t_LR _*as shown by the solid curve in Figure 4(b). As the polarization of the leads is fixed, both spin-up and spin-down line-width functions will be enhanced with increasing *t_LR_*, resulting in increased total current as shown in Figure 4(a). For the antiparallel case (*φ *= *π*), the current magnitude will also be enhanced for the same reason. But the current spin polarization is irrelevant to the tunnel process through the bridge since *ρ*_*L*↑ _= *ρ*_*R*↓ _= *ρ*_0 _(1 + *P_β_*) and *ρ*_*L*↓ _= *ρ*_*R*↑ _= *ρ*_0 _(1 - *P*_*β*_). The inter-lead line-width functions of both spin components are equal . The current spin polarization is mainly determined by the transport process through the QD. From the above discussion we also know that the current magnitude of the parallel configuration through the bridge is larger than that of the antiparallel alignment. With the increase of *t_LR_*, current through the bridge play a dominant role as compared with that through the dot, and the reverse spin valve effect may emerge accordingly. For the case of 0 <*φ < π*, the behavior of the current can also be understood with the help of the above discussions. Due to the reverse spin valve effect, the TMR in Figure 4(c) is reduced with increasing *t_LR_*, and becomes negative for high enough inter-lead coupling strength.

## Conclusion

We have studied the characteristics of tunneling current, current spin polarization and TMR in a quantum-dot-ring with noncollinearly polarized magnetic leads. It is found that the characteristics of these quantities can be well tuned by the relative angle between the leads' magnetic moments. Especially in the Coulomb blockade and strong inter-lead coupling strength range, the currents of larger angles are larger than those of smaller ones. This phenomenon is quite different from the usual spin-valve effect, of which the current is monotonously suppressed by the increase of the angle. The TMR in this range can be suppressed even to negative, and the current spin polarizations of parallel and antiparallel configurations individually approach to unit and zero, which can then serve as a effective spin filter even for usual ferromagnetic leads with 0 <*P_β _*< 1.

## Competing interests

The authors declare that they have no competing interests.

## Authors' contributions

JMM and JZ carried out numerical calculations as well as the establishment of the figures. KCZ, YJP and FC established the theoretical formalism and drafted the manuscript. FC conceived of the study, and participated in its design and coordination.
